# The Path Planning Problem of Robotic Delivery in Multi-Floor Hotel Environments

**DOI:** 10.3390/s25061783

**Published:** 2025-03-13

**Authors:** Linghui Han, Junzhe Ding, Songtao Liu, Meng Meng

**Affiliations:** 1 School of Maritime Economics and Management, Dalian Maritime University, Dalian 116026, China; junzhe_ding@outlook.com (J.D.);; 2 School of Management, University of Bath, Bath BA2 7AY, UK; mm3042@bath.ac.uk

**Keywords:** robot, path planning, multi-floor hotel, adaptive large neighborhood search

## Abstract

Robots have been widely adopted in transportation and delivery applications. Path planning plays a critical role in determining the performance of robotic systems in these tasks. While existing research has predominantly focused on path planning for single robots and the design of robot delivery systems based on hotel-specific demand characteristics, there is limited exploration of multi-robot collaborative routing in three-dimensional environments. This paper addresses this gap by investigating the multi-robot collaborative path planning problem in three-dimensional, multi-floor hotel environments. Elevator nodes are modeled as implicit waypoints, and the routing problem is formulated as a Multi-Trip Vehicle Routing Problem (MTVRP). To solve this NP-hard problem, an Adaptive Large Neighborhood Search (ALNS) algorithm is proposed. The effectiveness of the algorithm is validated through comparative experiments with Gurobi, demonstrating its ability to handle complex three-dimensional delivery scenarios. Numerical results reveal that the number of robots and elevator operation times significantly impact overall delivery efficiency. Additionally, the study identifies an imbalance in resource utilization, where certain robots are overused, potentially reducing their lifespan and affecting system stability. This research highlights the importance of efficient multi-robot routing in three-dimensional spaces and provides insights into optimizing delivery systems in complex environments.

## 1. Introduction

Robots have become increasingly integral to transportation and delivery applications due to their efficiency and flexibility. In the hospitality industry, robots are widely deployed for tasks such as food and item delivery, as seen in hotels like Japan’s Henn-na Hotel, Hangzhou’s FlyZoo Hotel, and Marriott Hotels in the United States [1]. These deployments not only enhance operational efficiency but also ensure safety, particularly during critical periods such as the COVID-19 pandemic, where robotic delivery minimized human contact, protecting both staff and guests from infection risks. Furthermore, robots alleviate repetitive labor burdens on hotel employees, allowing human workers to focus on higher-value services that require emotional intelligence and personalized care. When properly designed with privacy-preserving mechanisms (e.g., anonymized location tracking and restricted data retention), robotic systems can mitigate concerns about guest privacy. Additionally, embedded collision avoidance algorithms and real-time monitoring ensure safe navigation in crowded corridors, addressing physical safety risks. Such human–robot collaboration frameworks demonstrate how ethical challenges can be proactively resolved through technical and operational safeguards. However, despite their potential, the full capabilities of robots in improving hotel services remain underutilized [2]. A key challenge lies in designing effective delivery routes that meet guest time requirements while minimizing total travel time, thereby optimizing overall service quality.

The delivery process for hotel robots typically begins at a central depot, often located on the first floor or in the kitchen, and involves transporting items to designated guest rooms across multiple floors. Due to limited robot capacity, multiple trips are often required to complete all delivery tasks. This scenario aligns with the Multi-Trip Vehicle Routing Problem (MTVRP), a well-studied problem in logistics and operations research [3]. The MTVRP allows vehicles to perform multiple trips within a planning period, departing from the depot to deliver to specific customer rooms and returning to the depot to commence subsequent trips. Over the past three decades, significant progress has been made in addressing the MTVRP. For instance, ref. [4] explored the MTVRP with time windows and release times, proposing an exact branch-and-price algorithm that incrementally generates feasible routes using column generation techniques. Similarly, ref. [5] developed an exact algorithm combining dynamic programming and column generation to address the MTVRP with time windows and limited duration constraints. Mingozzi et al. [6] proposed a set-partitioning-based exact algorithm that decomposes the problem into smaller subproblems and applies column generation with valid inequalities. In terms of heuristic approaches, ref. [7] introduced an Adaptive Memory Programming method to improve the efficiency of solving large scale MTVRPs. Recently, some researchers have proposed variants of MTVRPs for real-world applications. For example, ref. [8] proposed a MTVRP with a time-dependent ready time function and duration function, named MT-TDVRPTW. The MT-TDVRPTW proposed by [8,9] introduced a split algorithm based on dynamic programming. Ref. [10] developed a post-processing greedy heuristic to solve a MT-TDVRPTW with soft time window and overtime constraints. Additionally, ref. [11] proposed a MT-VRPTW with unloading queues and designed an exact branch-and-price-and-cut algorithm, significantly improving the efficiency of solving such problems.

While significant progress has been made in solving the MTVRP in two-dimensional spaces, hotel environments introduce additional complexity due to their multi-floor, three-dimensional structures. Planning delivery routes in such environments requires not only optimizing horizontal movements but also coordinating vertical transitions, such as elevator usage, which significantly impacts delivery efficiency. Existing research on robot routing primarily focuses on single-robot path planning in two-dimensional environments [12,13,14] or system design based on demand predictions [15,16]. However, these approaches do not address the challenges of multi-robot collaborative path planning in three-dimensional spaces, particularly in complex multi-floor hotel settings.

This paper bridges this gap by investigating the multi-robot collaborative path planning problem in three-dimensional hotel environments. Elevator nodes are modeled as implicit waypoints, and the problem is formulated as a MTVRP. Given the NP-hard nature of the problem, an Adaptive Large Neighborhood Search (ALNS) algorithm is proposed to optimize delivery routes for multiple robots.

The contributions of this study are threefold. First, it extends the classic MTVRP to three-dimensional environments, addressing the unique challenges of multi-floor delivery. Second, it proposes an efficient ALNS algorithm to solve this complex problem, validated through comparative experiments with Gurobi. Third, it provides insights into the impact of robot allocation and elevator scheduling on delivery efficiency, highlighting the importance of balanced resource utilization in multi-robot systems.

The remainder of the paper is organized as follows. Section 2 presents the notations and mathematical model for the multi-floor robot delivery problem. Section 3 introduces the ALNS algorithm. Section 4 validates the algorithm through numerical experiments and analyzes key factors affecting delivery efficiency. Finally, Section 5 concludes the paper with remarks and future research directions.

## 2. Problem Description and Notations

**Figure 1:**
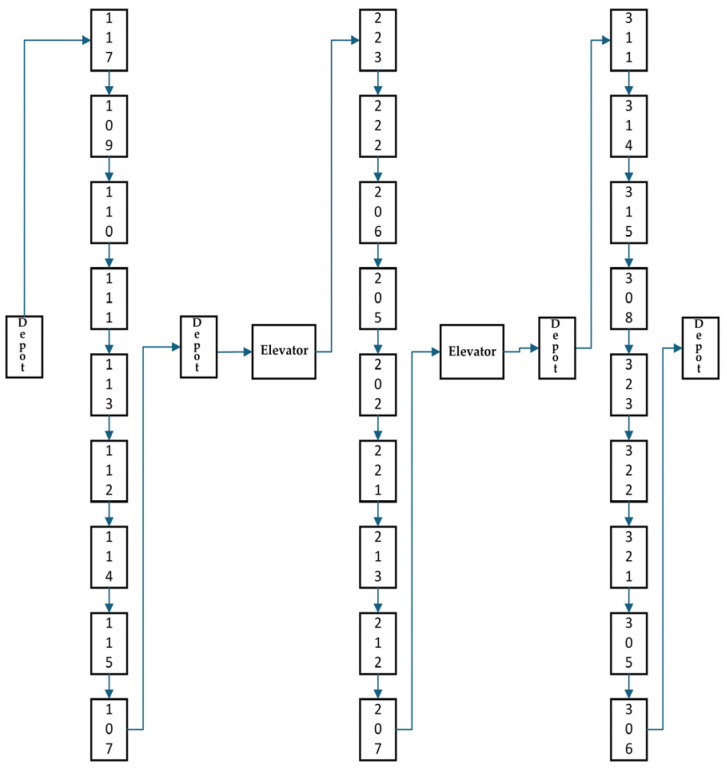
Example of Hotel Robot Delivery Routes.

In this study, a single hotel robot delivery trip is segmented into the following steps after collecting the items: (1) moving from the pickup location to the elevator, (2) taking the elevator to the floor of the customer’s room for delivery, and (3) repeating step (2) until all items from the current pickup are delivered. It should be noted that step (2) includes both same-floor and multi-floor deliveries, depending on the customer’s room location. Then, the robot returns to the pickup node to collect the remaining items for the next delivery trip. This delivery process can be illustrated by Figure 1. During a single delivery trip, each customer node is visited once, while the pickup node is accessed twice. Each elevator node of related floors is visited two times, once for traveling up and once for traveling down. The time required for a robot to travel from one floor to another via an elevator comprises two key components: the elevator waiting time and the elevator travel time. The elevator travel time can be estimated as a fixed value, determined by the building’s elevator speed and the vertical distance between floors. In real-world scenarios, the elevator waiting time on a particular floor is variable due to the influence of other passengers boarding the elevator on different floors. This variability can be modeled based on historical data collected over different time periods. Its mean value serves as a reasonable and practical estimate. Therefore, this study utilizes the average elevator waiting time for each floor as the estimated time a robot spends waiting for the elevator.

Beyond the elevator operation assumptions, two additional simplifications ensure computational tractability:

(1) Constant-speed robot operation with no battery constraints: This reflects short-duration hotel deliveries, where task completion aligns with typical battery lifespans (e.g., 8-h shifts per charge [1,2]), avoiding energy management complexities that dominate long-haul logistics.
(2) Static demand and time windows: Orders are assumed fixed during planning, consistent with batch-processing practices in hospitality operations (e.g., meal delivery waves). While dynamic requests occur in practice, this baseline assumption isolates routing efficiency analysis from real-time replanning challenges.

Based on these assumptions, this study formulates the robotic delivery process in multi-floor hotels as a MTVRP. The framework abstracts the hotel environment into a graph, where customer nodes are interconnected via spatiotemporal edges, embedding elevator transitions as implicit routing costs that combine fixed travel times and floor-specific waiting delays. Within this MTVRP paradigm, each robot’s multi-trip operations are constrained by capacity limits and time windows, while the objective function minimizes total delivery time through coordinated scheduling of horizontal movements and vertical transitions. To solve this problem, the framework integrates an ALNS algorithm that iteratively refines routes through adaptive destruction–repair cycles, dynamically balancing exploration of diverse route configurations and exploitation of high-efficiency paths. Validation is conducted through comparative benchmarking against exact solvers and parametric sensitivity analyses, quantifying the impact of key factors such as robot fleet size and elevator efficiency.

As mentioned in the research framework, we map the hotel robot delivery problem to an undirected graph G=(V,A), where V={0,…,n,n+1} represents the set of all nodes, with 0 denoting the starting depot, n+1 denoting the return depot, and $V^{\prime }=\{1,\ldots ,n\}$ representing the set of all customer room nodes requiring delivery. Let $V^{+}=\{0,\ldots ,n\}$ and $V^{-}=\{1,\ldots ,n+1\}$; the edge from node i to node j is denoted by (i,j), and the set of all edges is defined as $A=\{(i,j):i\in \{0\},j\in V^{\prime },i\in V^{\prime },$ $j\in V^{-},i\neq j\}$. Here, $\delta^{-}(i)$ and $\delta^{+}(i)$ represent the predecessor and successor nodes of node i, respectively. Each customer node has a demand $d_{i}\geq 0$ and a time widow $\left[a_{i},b_{i}\right]$. Assume the hotel employs K robots, each with a maximum capacity of Q. Each robot can undertake multiple trips, servicing several customer nodes during each trip. The robot’s speed is assumed to be its average operating speed, treated as a constant, with each arc (i,j) associated with a specific travel time $t_{ij}$. This study defines the service time for each customer node $i\in V^{\prime }$ as $e_{i}$, where $t_{ij}$ represents the robot’s travel time from node i to node j. The parameters and variables in the MTVRP model are as follows:

(1) Parameters:

Q: The robot’s maximum capacity

$d_{i}$: The demand for custom node i,

$t_{ij}$: The time required for delivery from node i to node j,

M: A constant that is sufficiently large,

K: The total number of robots.

(2) Variables:

$x_{ij}$: Equals the value 1 if the robot’s delivery route includes arc $\left(i,j\right)$; otherwise, 0,

$z_{ij}$: Equals 1 if a delivery concludes at customer node i and a subsequent delivery begins at the customer node j; otherwise, 0,

$q_{ij}$: represents the weight of goods passing through arc,

$\tau_{j}$: represents the service start time (in seconds) for customer node.

(3) Variable domains:

$x_{ij}\in \{0,1\},\forall (i,j)\in A$,

$z_{ij}\in \{0,1\},\forall (i,j)\in A$,

$q_{ij}\geq 0,\forall (i,j)\in A$,

$\tau_{i}\geq 0,\forall i\in V^{-}$.

Based on the defined variables and parameters, the MTVRP model for the hotel robot delivery problem in this study is formulated as follows:

(1) $$\min\sum_{(i,j)\in A}t_{ij}x_{ij}$$

(2) $$\sum_{j\in \delta^{+}(i)}x_{ij}=1,\;\forall i\in V^{+},$$

(3) $$\sum_{j\in \delta^{-}(i)}x_{ji}=1,\;\forall i\in V^{+},$$

(4) $$\sum_{j\in \delta^{+}(0)}x_{0j}=\sum_{j\in \delta^{-}(n+1)}x_{j,n+1},$$

(5) $$\sum_{i\in \delta^{-}(j)}q_{ij}-\sum_{i\in \delta^{+}(j)}q_{ji}=d_{j},\;\forall j\in V^{\prime },$$

(6) $$q_{ij}\leq Qx_{ij},$$

(7) $$q_{i,n+1}=0,\;\forall i\in V^{\prime },$$

(8) $$\tau_{i}+e_{i}+t_{ij}-M(1-x_{ij})\leq \tau_{j},\;\forall i\in V^{\prime },j\in V^{-},$$

(9) $$a_{i}\leq \tau_{i}\leq b_{i},\;\forall i\in V^{-},\;$$

(10) $$\sum_{\begin{array}{l} i\in V^{\prime }\\ i\neq j \end{array}}z_{ij}\leq x_{0j},\;\forall j\in V^{\prime },\;$$

(11) $$\sum_{\begin{array}{l} j\in V^{\prime }\\ j\neq i \end{array}}z_{ij}\leq x_{i,n+1}\;\forall i\in V^{\prime },$$

(12) $$\tau_{i}+\left(t_{i,n+1}+t_{0j}\right)\leq \tau_{j}+\left(1-z_{ij}\right)M\;\;\forall i,j\in V^{\prime },i\neq j,$$

(13) $$\sum_{j\in V^{\prime }}x_{0j}-\sum_{i\in V^{\prime }}\sum_{\begin{array}{l} j\in V^{\prime }\\ j\neq i \end{array}}z_{ij}\leq K.$$

The objective function (1) of this model minimizes the total travel time. The binary variable $x_{ij}$ determines the robot’s routing, where $x_{ij}$ indicates traversal of arc (i,j). Constraints (2) and (3) enforce unique customer visits, while constraint (4) balances depot flows, collectively forming a 3D routing network with elevator costs embedded in arc weights. The variable $q_{ij}$ coupled with $x_{ij}$ via constraint (6), simultaneously tracks payload distribution and enforces capacity limits, while constraint (5) eliminates subtours through flow conservation. Constraint (7) ensures that the robot does not return goods to the depot. Temporal coordination is governed by $\tau_{i}$, where constraint (8) establishes precedence relationships between nodes using big M relaxation, and constraint (9) enforces strict time windows. Spatio–temporal coupling emerges from constraints (8) and (9), where elevator waiting times (via $t_{ij}$) and hard time windows jointly model resource contention in hotel environments. The trip transition variable $z_{ij}$ coordinates multi-trip operations. If $z_{ij}=1$ enforces that after completing delivery at i, the robot returns to the depot and initiates a new trip to j, with constraint (12) ensuring temporal continuity between consecutive trips. Multi-robot coordination is achieved through constraint (13), which dynamically allocates trips by regulating robot deployments $\sum x_{0j}$ and trip transitions $\sum z_{ij}$.

## 3. Algorithm

The multi-floor hotel robot delivery problem studied in this research is an extension of the MTVRP. For larger problem scales, heuristic algorithms are essential to find an acceptable solution. The ALNS algorithm is widely recognized for solving complex Vehicle Routing Problems (VRP) and their extensions, such as multi-trip and time-constrained variants. It iteratively improves solutions through adaptive destruction and repair operations, guided by a dynamic scoring mechanism, allowing it to escape local optima while refining high-quality solutions. Therefore, this study develops an ALNS-based algorithm (Algorithm 1) for the model (1)–(13). First, this section presents its framework and then provides a detailed description of the algorithm’s design.
**Algorithm 1**: ALNS algorithm frameworkInput: Customer nodes, robot fleet, elevator constraints Output: Optimized delivery routes 1. Initialize routes using Savings Algorithm and 2-opt 2. While (temperature > min_temperature) and (iterations < max_iterations):       a. Select removal operator (roulette wheel) → Remove q nodes       b. Select insertion operator (roulette wheel) → Reinsert nodes       c. Evaluate new solution (total travel time)       d. Update operator scores/weights adaptively       e. Apply simulated annealing: Accept/reject solution 3. Return best solution 

**(1) Initialization**

First, all customer nodes are grouped according to their respective floors. For each floor, the Savings Algorithm [17] is used to generate initial delivery routes. During this process, customer demands that exceed the capacity of a single robot are reassigned to a new delivery robot. Subsequently, the 2-opt algorithm is applied to locally optimize the generated delivery route, reducing the route length and improving delivery efficiency.

During the route generation process, for each customer node, the algorithm first calculates the cost of the robot completing the delivery on the same floor. If the customer nodes are on different floors, the robot must use the elevator to perform deliveries across floors. In such cases, the route cost must include the elevator waiting time and travel time. For each customer node, the algorithm calculates the incremental cost of adding the node to the current route and selects the position with the smallest incremental cost for insertion.

After all customer nodes are assigned to suitable routes, the algorithm checks for any unmet constraints, such as capacity or time window limitations. If any customer nodes cannot be assigned to existing routes due to these constraints, a new robot is introduced, and a new route is assigned to ensure all customer nodes are satisfied. Finally, the algorithm generates a preliminary feasible solution, laying a robust foundation for subsequent global optimization.

**(2) Removal Operators**

After generating the initial solution, the algorithm performs local operations on the current solution by designing various removal operators. The selection of removal operators is determined by a roulette wheel mechanism, which assigns a probability to each operation based on its effectiveness, allowing for a probabilistic selection of the operators. The specific designs of the removal operators are as follows:

After generating the initial solution, the algorithm performs local operations on the current solution by designing various removal operators. The selection of removal operators is determined by a roulette wheel mechanism, which assigns probabilities to each operation based on its effectiveness, allowing for a probabilistic selection. Each operation removes several customer nodes from the current solution, producing a partial solution S and a set q of removed customer nodes. The specific designs of the removal operators are as follows:

(a) Random removal: Randomly select q customer nodes for removal.
(b) Worst removal: Calculate the contribution of each customer node to the total travel time, where the contribution is defined as the difference between the total travel time after removing the customer node and the original total travel time. Remove q customer nodes with the highest contributions.
(c) Floor-related removal: Randomly select a floor and prioritize removing customer nodes belonging to that floor. If the number of customer nodes on the floor is less than required q, randomly select additional customer nodes from other floors to meet the quota.
(d) Time window-related removal: Sort customer nodes by the midpoint of their time windows, randomly select a seed customer node i, and remove the q−1 customer nodes whose time window midpoints are closest to that of i.

**(3) Insertion Operators**

After the removal operator operation, the algorithm reinserts the removed q customer nodes into the robot’s delivery path. The selection of insertion operators is also determined randomly using a roulette wheel mechanism. During the insertion process, if no remaining customer nodes can satisfy the current path constraints, a new robot is introduced, and a new route is created for it. This continues until all customer nodes are successfully inserted. The specific designs of the insertion operators are as follows:

(a) Greedy insertion: For each customer node to be inserted, calculate the increase in total travel time for each feasible insertion position in the robot’s current delivery path and select the position with the smallest increase for insertion.
(b) Regret-k insertion [18]: Calculate the k best insertion positions for each customer node to be inserted and compute the regret value:

$$regret(i)=\sum_{j=2}^{k}\;\left(c_{ij}-c_{i1}\right)$$

where $c_{ij}$ is the delivery time of the jth best insertion position. The node with the largest regret value is chosen for insertion.

(c) Floor-priority insertion: Group the customer nodes to be inserted by their floor and prioritize inserting them into paths on the same floor. If same-floor insertion is infeasible or results in excessive delivery time, consider cross-floor insertion.
(d) Time window-related insertion: Sort the customer nodes to be inserted by their time window midpoints, prioritize those with tighter time windows, assess the impact on path feasibility post-insertion, and select the insertion position that minimizes time window violations.

**(4) Adaptive Layer Scoring Mechanism**

One of the key strengths of the ALNS algorithm is its adaptive mechanism, which enables it to learn and adjust the weights and scores of the operators throughout the search process. The adaptive process in this study is designed as follows:

Step 1: Weight initialization: Initialize the weight $w_{i}$ and score $\pi_{i}$ for each operator i.

Step 2: Score update: In each iteration, update the score of the operators based on their performance:

If a new global optimal solution is found, $\pi_{i^{+}}=\sigma_{1}$.

If a better solution is found, $\pi_{i^{+}}=\sigma_{2}$.

If an accepted solution is found, $\pi_{i^{+}}=\sigma_{3}$.

Here, $\sigma_{1}>\sigma_{2}>\sigma_{3}$.

Step 3: Weight update: After every iteration, update the weight of the operator i as: $w_{i}=\left(1-r\right)w_{i}+r\;\pi_{i}/\theta_{i}$, where r is the reaction factor, and $\theta_{i}$ is the usage count of operator i.

Step 4: Reset scores: Set and reset the usage count $\theta_{i}=0$.

To balance the algorithm’s exploration ability and convergence speed, this study adopts the Simulated Annealing (SA) mechanism as the solution ac-acceptance criterion [19]. The specific design is as follows:

(1) Accept better solutions: If the new solution s′ is better than the current solution s, the new solution is always accepted.
(2) Accept worse solutions: If the new solution s′ is worse than the current solution, it is accepted with probability: $exp^{-\left[f\left(s\prime \right)-f\left(s\right)/T\right]}$, where $f\left(s\prime \right)$ is the objective function value of the new solution, and T is the current temperature. The temperature T gradually cools according to T=T⋅η, where η is the cooling rate.

By incorporating the simulated annealing mechanism, the algorithm can accept worse solutions in the early stages, escape local optima, and enhance global optimization performance. In the case analysis section of this paper, the designed algorithm is compared with the Gurobi solver to validate its effectiveness.

## 4. Numerical Tests

The ALNS algorithm proposed in this study is implemented in Python 10, and the mathematical model is constructed and solved using Gurobi 11.0.0. The experiments were conducted on a computer equipped with an Intel (R) Core (TM) i7-10700K CPU @ 3.80 GHz, 32 GB RAM, and running the Windows 10 operating system. The distance matrix used in the experiments is derived from the actual room layout data of a hotel in China.

To validate the effectiveness of the proposed ALNS algorithm for the multi-floor hotel robot meal delivery problem, this study designed and conducted multiple sets of numerical experiments. In the experiments, the results of the ALNS algorithm are first compared with those of Gurobi to assess its feasibility and performance advantages. Subsequently, the study further analyzed the impact of various parameters on the delivery results, specifically including:

(1) The impact of different delivery demand quantities on delivery performance under the same number of robots and elevator operation times, including elevator waiting time and elevator travel time.
(2) The impact of different numbers of robots on delivery performance under the same demand quantity and elevator operation times, including elevator waiting time and elevator travel time.
(3) The impact of different elevator operation times, including elevator waiting time and elevator travel time on delivery performance under the same demand quantity and the numbers of robots.
(4) The impact of different demand quantities, numbers of robots, and elevator operation times, including elevator waiting time and elevator travel time on the number of robot trips $z_{oj}$.

### 4.1. Algorithm Parameters and Case Description

Based on the research on ALNS algorithm parameter settings in the existing literature and considering the unique aspects of multi-floor hotel robot meal delivery, this study adopts the parameter setting strategies proposed by Ropke, Masson et al. [20,21] with the following parameters: The number of destruction operators q=6, and the number of repair operators r=5. The maximum proportion of customer points removed in each iteration is set to 0.4 of the total customer points. This proportion ensures sufficient search space for the algorithm without causing excessive computation time. The noise parameters are set to $p_{n}=0.1$ and $\eta_{n}=0.025$, which helps the algorithm escape local optima.

Regarding the parameters of the adaptive layer scoring mechanism in the algorithm: The scores for accepting solutions are set to $\sigma_{1}=33,\;\sigma_{2}=20,\;\sigma_{3}=10$. The score update weight ratio ρ=0.1. The cooling coefficient η of the algorithm is set to 0.95 to ensure a sufficiently slow cooling rate. For the solution acceptance probability parameters, the initial temperature $T_{0}=1000$, where ω=0.05 and $c_{0}$ is the cost of the initial solution.

**Figure 2:**
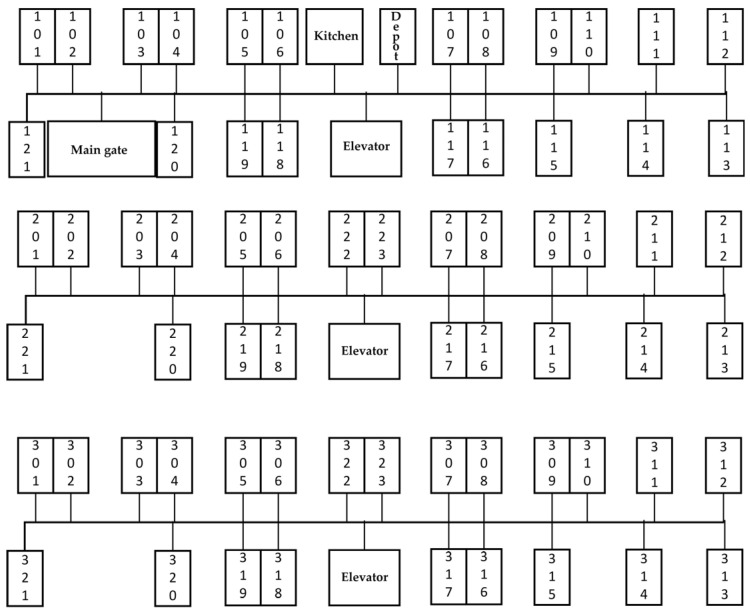
Layout of Hotel Rooms.

This study conducts numerical experiments based on an actual hotel scenario with a total of 67 rooms. The layout is shown in Figure 2, where the second and third floors have the same layout. The room numbers and floor distribution are as follows:

First floor: Rooms numbered 1 to 21, totaling 21 rooms.

Second floor: Rooms numbered 22 to 44, totaling 23 rooms.

Third floor: Rooms numbered 45 to 67, totaling 23 rooms.

The distances between floors and rooms in the hotel are derived from the actual hotel floor plan and floor height, resulting in a 68 × 6868 × 68 distance matrix (including the meal depot 0 and 67 rooms).

**Figure 3:**
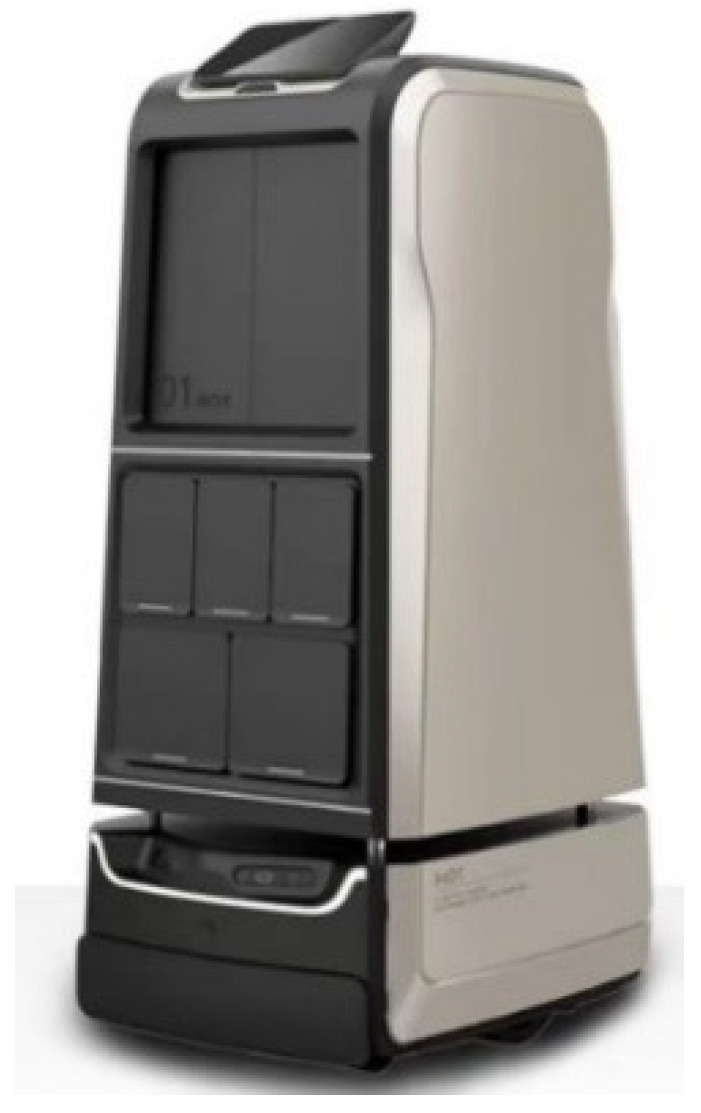
The Multi-cabin Hotel Delivery Robot.

To simulate different delivery demand scenarios, this study uses random sampling to generate varying numbers of meal delivery requests:

(1) Demand quantity setting: The study defines 10, 20, 30, and 60 customer nodes requiring delivery services, representing various scales of delivery scenarios.
(2) Selection of customer nodes: For each experimental scenario, a corresponding number of rooms are randomly selected from all rooms as the nodes requiring delivery, ensuring the fairness and randomness of the experiments.
(3) Setting of customer demand: For each selected room, the delivery demand is randomly generated, ranging from one to three units, simulating potential meal orders or other items from guests while adhering to the capacity constraints of a single robot delivery.
(4) Robot capacity setting: Each delivery robot has a maximum capacity of 12 units, consistent with the load limitations of real-world robot devices. The delivery robot used in the case analysis of this study is illustrated in Figure 3.
(5) Elevator operation time: Since elevator operation time, including elevator waiting time and elevator travel time, plays a critical role in multi-floor de-livery processes, but the elevator waiting time in practice is affected by random factors such as passenger traffic and usage frequency, this study sets the base waiting time for elevators at 30 s. Additionally, varying elevator operation times are defined based on different time periods (peak and off-peak), ranging from a minimum of 40 s to a maximum of 100 s, to examine the effect of elevator operation time on delivery outcomes.

### 4.2. Algorithm Validation

To verify the feasibility and effectiveness of the proposed ALNS algorithm, this study compares it with the commercial optimization solver Gurobi.

In the experiments, this study uses different scales of customer nodes (10, 20, 30, 40, 50, 60 customer nodes) and designs two special scenarios: demand rooms concentrated on higher floors and demand nodes concentrated on lower floors.

By comparing the ALNS algorithm of this study with Gurobi, it is observed that for a scenario with 20 customer nodes, Gurobi requires the computation of a complex model involving over 3000 variables and constraints. In cases with more than 30 customer nodes, Gurobi’s running time increases significantly, leading the authors to set a maximum running time of 18,000 s in this study. Table 1 presents the solving time, results obtained, and the GAP between the results of Gurobi and the ALNS algorithm, where GAP = (ALNS Solution − Gurobi Solution)/Gurobi Solution × 100% and average percentage deviation across 10 experimental runs.

From the experimental results in Table 1, it is evident that the ALNS algorithm matches Gurobi’s solutions exactly (GAP of 0%) for small-scale problems (e.g., 10 and 20 customer nodes), with the ALNS algorithm having an extremely short solving time. For instance, in the scenario with 10 customer nodes, Gurobi’s solving time is 1.55 s, while the ALNS algorithm takes only 0.01 s. In the scenario with 20 customer nodes, Gurobi’s solving time is 600.21 s, compared to 0.02 s for the ALNS algorithm.

The exact match in solutions for small-scale problems underscores the efficiency of the ALNS algorithm in addressing such problems.

As the problem size increases, particularly in medium-scale scenarios (e.g., 30 customer nodes), Gurobi’s solving time rises sharply to 18,000 s, while the ALNS algorithm takes only 0.09 s, demonstrating a clear advantage over Gurobi. In this scenario, the gap between the ALNS algorithm’s solution and Gurobi’s exact solution is only 0.64%. This indicates that while ALNS produces solutions quickly, the quality of these solutions is very close to Gurobi’s solutions, with acceptable deviations.

For large-scale problems (e.g., 40, 50, and 60 customer nodes), Gurobi’s solving time also reaches 18,000 s, whereas the ALNS algorithm’s solving times are 4.50 s, 8.70 s, and 14.15 s, respectively, showcasing a significant computational speed advantage. However, as the problem size grows further, the difference between the ALNS algorithm’s solutions and Gurobi’s solutions widens, with GAPs of 1.40%, 1.43%, and 0.41%, respectively. This phenomenon aligns with the study’s experimental expectations, as a large-scale problem typically results in greater deviations between ALNS’s quick solutions and the optimal solutions. Nevertheless, even in large-scale problems, the solutions generated by ALNS remain within a reasonable error range, providing efficient solutions for practical applications. Notably, with the optimal parameter combination—60 customer nodes, eight robots, and an elevator operation time of 40 s—the GAP is only 0.41%, demonstrating that the algorithm can still achieve high-quality solutions for large-scale problems involving multiple customer nodes and robots.

In scenarios with high-floor and low-floor customer nodes, the difference between ALNS and Gurobi’s solutions is minimal, particularly in the low-floor scenario, where the GAP is 0%. This indicates that the ALNS algorithm can effectively handle special customer demand distributions, as well as Gurobi in these scenarios. Similarly, the ALNS algorithm’s solving time in these scenarios is significantly shorter than Gurobi’s, highlighting its clear time advantage.

Overall, the ALNS algorithm exhibits notable time advantages across all problem sizes. Particularly when the number of customer points exceeds 30, Gurobi’s solving time increases sharply, while the ALNS algorithm can still produce high-quality solutions in a relatively short time. Although minor discrepancies exist between the ALNS algorithm’s solutions and Gurobi’s optimal solutions in large-scale problems, its solving time is considerably shorter than Gurobi’s, validating the efficiency and feasibility of the ALNS algorithm designed in this study for tackling large-scale, multi-constraint problems.

### 4.3. Analysis of Delivery Performance Impact

To systematically analyze the key factors affecting robot delivery efficiency in multi-floor hotel scenarios, this study employed orthogonal experiments to investigate the impact of three parameters—number of customer nodes, number of robots, and elevator operation time—on the total travel time. The experimental results reveal the interactions between these factors and their specific effects on delivery performance. This section provides a detailed presentation and analysis of the experimental results.

The orthogonal experiments are designed using an L36 (6 × 8 × 7) experimental table, systematically examining variations in delivery efficiency under different combinations of the three factors: number of customer nodes, number of robots, and elevator operation time. The main parameters and levels configured in the experiments are as follows:

Number of customer nodes: 10, 20, 30, 40, 50, 60.

Number of robots: 1, 2, 3, 4, 5, 6, 7, 8.

Elevator operation time: 40, 50, 60, 70, 80, 90, 100 s.

The experimental results are assessed using the total travel time (in seconds), with the average of 10 runs calculated for each group. Table 2 presents some orthogonal experimental combinations and their corresponding total delivery time results:

(1) Analysis of the impact of the number of customer nodes on delivery performance

**Figure 4:**
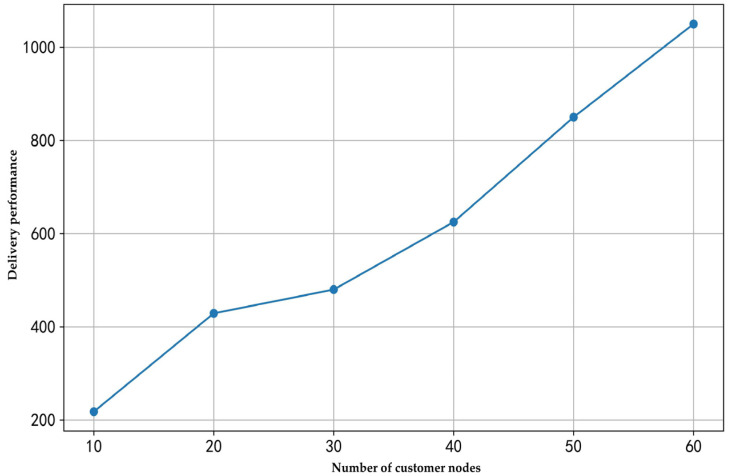
The impact of the number of customer nodes on delivery performance.

To analyze how the total delivery cost changes with the number of customer nodes under the same number of robots, this study conducted experiments with one robot and an elevator operation time of 40 s for scenarios involving 10, 20, 30, 40, 50, and 60 rooms. The experimental results are illustrated in Figure 4.

As shown in Figure 4, as the number of customer nodes increases, the total travel time exhibits a distinct linear growth trend. This indicates that with a fixed number of robots, as the volume of delivery tasks increases, robots must execute more round trips, causing the total travel time to rise. The greater the number of rooms, the heavier the workload on the robots, leading to a linear increase in delivery time.

(2) Analysis of the impact of the number of robots on delivery performance

**Figure 5:**
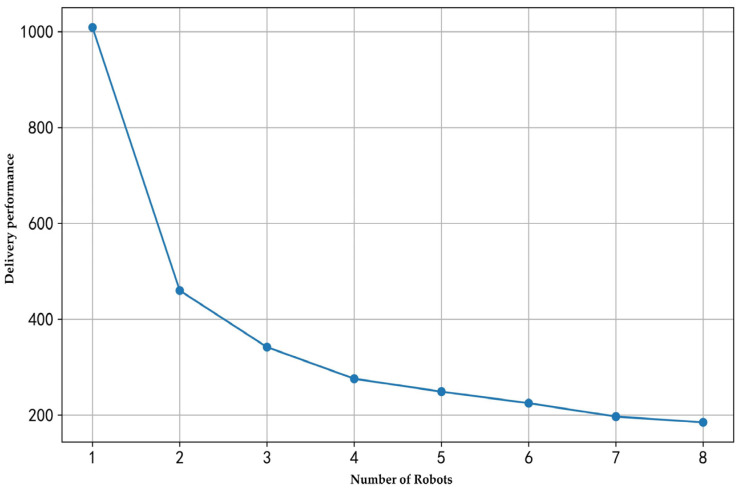
The impact of the number of robots on delivery performance.

To study the impact of different numbers of robots on delivery results under the same demand conditions, this study tested the delivery performance of one to eight robots with 60 customer nodes and an elevator operation time of 40 s. The experimental results are illustrated in Figure 5.

It is evident that as the number of robots increases, the total travel time decreases significantly. This is because more robots can distribute the delivery tasks, reducing the workload for each robot. However, the diminishing marginal effect is noticeable, as the efficiency improvement becomes less significant when the number of robots reaches five or more. For instance, in the scenario with 60 rooms, increasing the number of robots from five to eight reduces the total travel time only from 242 s to 225 s.

(3) Analysis of the impact of elevator operation time on delivery performance

**Figure 6:**
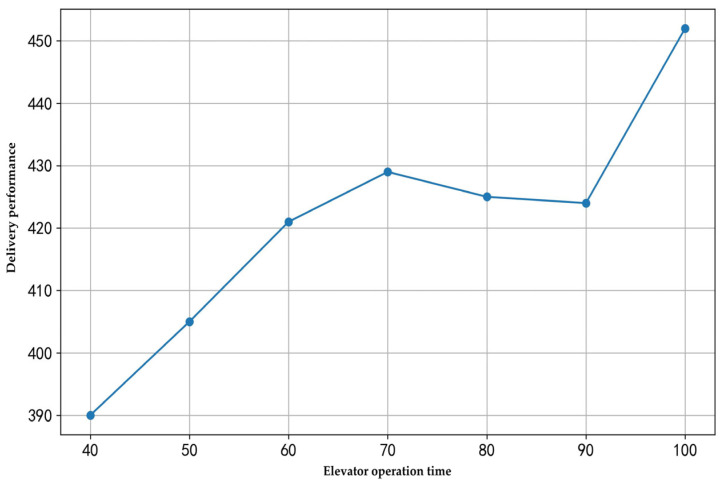
The impact of elevator operation time on delivery performance.

In multi-floor hotel delivery scenarios, the operation time of elevators significantly impacts delivery efficiency. To analyze the effect of elevator operation time on total travel time, this study conducted experiments with 60 rooms and three robots, using elevator operation times of 40, 50, 60, 70, 80, and 100 s. The experimental results are illustrated in Figure 6.

As shown in Figure 6, under the same demand and number of robots, different elevator operation times significantly affect delivery performance. As the elevator operation time increases, the total travel time exhibits a fluctuating upward trend. This indicates that the frequency of elevator usage and its operation time directly influence the efficiency of multi-floor delivery. Optimizing the delivery sequence of robots and minimizing cross-floor trips can effectively reduce the total travel time. This is particularly evident when the elevator operation time is extended, as its impact on the total travel time becomes notably significant.

(4) Analysis of the impact of three parameters on the number of robot trips

In this study, the term “number of robot trips” $z_{0j}$ refers to the count of times each robot departs from the depot, completes a sequence of delivery tasks, and returns to the depot. This metric not only directly influences the task load assigned to each robot during delivery but also significantly impacts the system’s total travel time. For the problem addressed in this paper, an increase in the number of robot trips necessitates repeated use of the elevator to return to the depot. Consequently, the frequency and duration of elevator operations significantly affect delivery efficiency. Then, the maximum number of trips executed by a single robot frequently becomes the system’s bottleneck. Therefore, analyzing variations in the number of robot trips is crucial to this study.

**Figure 7:**
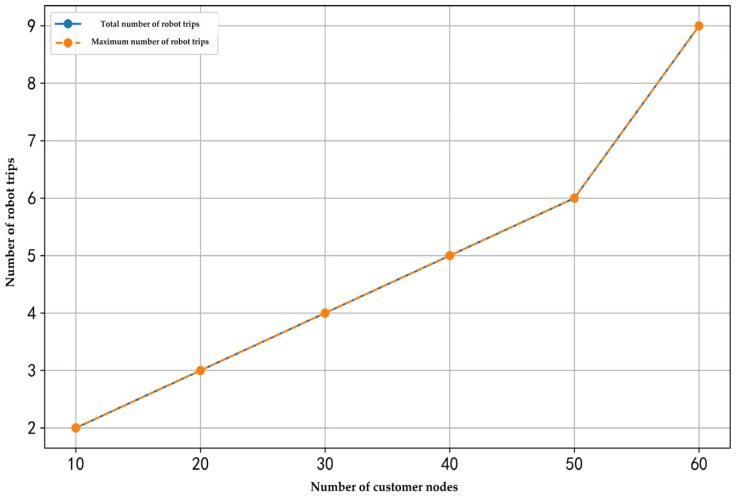
Effect of the number of customer nodes on the number of robot trips.

**Figure 8:**
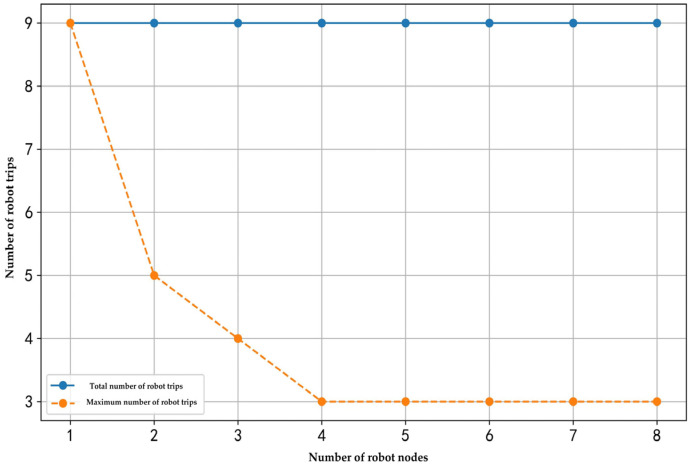
The effect of the number of robots on the number of robot trips.

**Figure 9:**
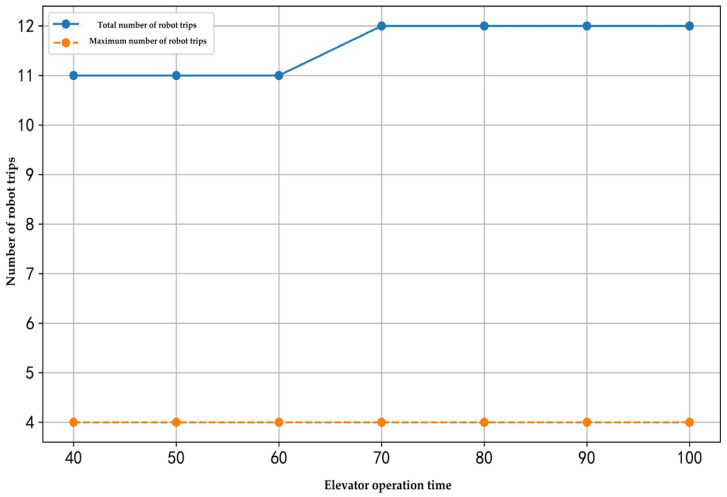
Effect of the elevator operation time on the number of robot trips.

This research examined the relationships between the number of customer nodes, the number of robots, elevator operation time, and the number of robot trips. The corresponding results are illustrated in Figure 7, Figure 8 and Figure 9.

As shown in Figure 7, when the number of robots and elevator operation time remain constant, and the number of customer nodes increases from 10 to 60, the total number of trips rises from two to nine. Under this experimental condition, where only one robot is deployed, the total number of trips equals the maximum number of trips executed by that robot. This finding demonstrates that, with a fixed number of robots and elevator operation time, an increase in the number of customer nodes requires the robot to perform more round trips to service all nodes, resulting in a linear growth in the number of robot trips.

As shown in Figure 8, when the number of customer nodes and elevator operation time remain constant, and the number of robots increases from one to eight, the total number of trips remains at nine. However, the maximum number of trips executed by a single robot decreases from nine to three. Notably, when the number of robots exceeds four, the reduction in the maximum number of trips gradually diminishes, and further increases in the number of robots have a limited impact on reducing the number of robot trips.

This finding demonstrates that, under a fixed number of customer nodes and elevator operation time, increasing the number of robots allows tasks to be distributed among more robots. As a result, the number of robot trips executed by a single robot gradually decreases, leading to more balanced task allocation and improved scheduling efficiency. However, as the number of robots increases, the diminishing marginal effect becomes evident, meaning that additional robots contribute less to further reducing the number of robot trips.

Figure 9 illustrates the relationship between elevator operation time and the number of robot trips. When the number of customer nodes and robots remains constant, and the elevator operation time increases from 40 to 100 s, the total number of trips remains between 11 and 12, while the maximum number of trips executed by a single robot stays around 4. This indicates that, although an increase in elevator operation time significantly extends the duration of each trip, it does not substantially alter the number of trips robots need to execute.

From the numerical experiment results, the number of robot trips not only determines the delivery burden of each robot but also directly influences the total travel time. Reducing the maximum number of trips executed by a single robot can significantly enhance the system’s overall scheduling efficiency. Furthermore, allocating trips reasonably among robots can balance task distribution and improve the flexibility and robustness of the scheduling strategy. Moreover, it can ensure efficient and stable system operation despite uncertainties such as task changes or equipment failures.

### 4.4. Discussion

From the numerical analysis, one can find that the relationship between the demand scale and the number of robots is critical for determining delivery efficiency. For scenarios with fewer demands (e.g., 10–20 rooms), 1–2 robots are sufficient to meet delivery needs. Deploying additional robots in such cases does not significantly improve efficiency and may lead to resource waste. As demand increases (e.g., 30–40 rooms), 3–5 robots are required to achieve higher delivery efficiency while maintaining reasonable costs. For large-scale demand scenarios (e.g., over 50 rooms), 6–8 robots are necessary to significantly reduce total travel time.

However, the marginal benefits of adding more robots diminish as their number increases. For instance, in the scenario with 60 rooms, increasing the number of robots from five to eight reduces the total travel time only from 242 s to 225 s, indicating a smaller efficiency gain. This suggests that while increasing the number of robots is necessary for large-scale demands, excessive deployment may lead to unnecessary costs. Therefore, dynamic adjustment of the number of robots should be a core consideration in delivery task planning.

Based on the results of numerical tests, elevator operation time is a significant bottleneck affecting multi-floor delivery efficiency, particularly during peak periods such as breakfast or check-out times, when elevator usage increases substantially. Experimental data shows that in the scenario with 60 customer nodes, increasing elevator operation time from 40 s to 100 s nearly doubles the total travel time (from 225 s to 500 s). Therefore, elevators act as critical bottlenecks in multi-floor delivery. Each cross-floor task requires robots to wait for and use elevators, and prolonged waiting/travel times (e.g., from 40 s to 100 s) directly increase the duration of every trip involving floor transitions. This underscores the limitations of traditional demand-driven robot scheduling during peak periods and highlights the need for optimized elevator scheduling and task allocation. To address this, the following strategies are recommended: (a) Implement one-way elevator operations (e.g., only up or only down) to reduce empty running time. (b) Assign high-priority elevator access to robots through scheduling rules, minimizing elevator operation time, and improving delivery efficiency.

From the numerical results, one can observe that while increasing the number of robots reduces total travel time, the marginal benefits decrease as more robots are added. For example, in large-scale demand scenarios (e.g., 60 customer nodes), increasing the number of robots from five to eight reduces delivery time, but the time saved per additional robot diminishes significantly. For hotel managers, deploying an excessive number of robots not only increases procurement and maintenance costs but may also result in idle resources. Therefore, identifying a “balance point” for the number of robots is essential to maximize resource allocation efficiency while meeting service quality requirements. For instance, in scenarios with high delivery demands (e.g., 50–60 nodes) and short elevator operation times (e.g., 40–60 s), configuring six robots achieves an optimal balance between efficiency and cost. During peak periods with longer elevator operation times, increasing the number of robots to 7–8 can significantly improve service quality and prevent customer dissatisfaction due to delays.

By the numerical result in Figure 8, an imbalance in robot utilization is observed under certain configurations. For instance, in scenarios with three robots servicing 60 customer nodes, one robot was assigned four delivery trips while the remaining two completed only two to three trips. Although the absolute difference appears modest in this case, the phenomenon suggests a systemic bias that could lead to some operational risks in large-scale applications. For example, overused robots (e.g., those with 50% more trips than peers) experience faster battery depletion and mechanical wear. Concentrated workloads increase the probability of simultaneous robot failures. Underutilized robots (e.g., those completing two trips vs. four) represent idle capital expenditure. This issue originates from the exclusive focus of our optimization model on total efficiency metrics without incorporating fairness terms (e.g., workload variance). Consequently, the ALNS algorithm naturally prioritizes solutions where certain robots are assigned “cost-effective” routes (e.g., clustered deliveries on the same floor with minimal elevator usage), while others remain under-deployed. This design choice aligns with classical MTVRP formulations but proves inadequate for sustainable multi-robot systems. Future work will address this gap by integrating equity-aware objectives, such as minimizing the maximum robot workload to achieve balanced utilization.

## 5. Conclusions

This study investigates the optimization problem of robot delivery in multi-floor hotel environments, considering specific customer demands and the number of robots available. The elevator, often used during the robot delivery process, is treated as an implicit node rather than a conventional delivery point in this study. The MTVRP model is used to model the robot delivery problem in multi-floor hotel environments, with an ALNS algorithm developed to solve it. The findings reveal that, for a fixed number of customer nodes, increasing the number of robots effectively reduces delivery time, though this effect diminishes as more robots are added. Although the elevator is not explicitly modeled as a node, its operational time has a significant impact on overall delivery efficiency. Furthermore, numerical experiments indicate that uneven usage frequency among robots in multi-robot systems can lead to issues such as reduced lifespans for frequently used robots and instability in the delivery system. This research provides both model and algorithmic support for optimizing robot delivery in multi-floor hotel environments.

The experimental results provide actionable insights for optimizing robotic delivery systems in multi-floor hotels. To balance efficiency and sustainability, demand-responsive robot deployment should be introduced to scale robot fleets dynamically. This can avoid over-provisioning-induced diminishing returns while leveraging flexible leasing models to address peak-hour surges (e.g., breakfast periods). Operational frameworks should integrate workload balancing constraints, such as enforcing per-robot trip limits (e.g., ≤4 trips/shift), to mitigate mechanical wear and systemic failure risks caused by imbalanced utilization. Furthermore, addressing vertical mobility bottlenecks requires elevator coordination strategies. For example, prioritizing robot-dedicated time slots during demand surges can reduce elevator waiting times and minimize empty returns via unidirectional scheduling.

Future research will explore several factors to better align with practical application scenarios, such as random or dynamic elevator operation times, dynamic demand fluctuations, and robot battery management for 24/7 operations or hospitals with continuous demand. Additionally, the models and algorithms developed in this study will be applied to other multi-floor delivery tasks, such as hospital ward supply management, mall restocking, and office building document deliveries. Future work will also investigate multi-robot collaboration mechanisms, collision avoidance mechanisms (e.g., velocity obstacles), intelligent elevator scheduling algorithms, and real-time optimization for dynamic demand scenarios. Our future research will rigorously analyze the trade-offs between compartment quantity, per-compartment capacity, and delivery performance, incorporating dynamic capacity allocation strategies. These advancements aim to foster the broader application of intelligent delivery technology across various domains.

## Figures and Tables

**Table 1:** Comparison of the ALNS Algorithm and Gurobi.

		Gurobi	Gurobi	ALNS	ALNS	GAP	Average G
Customer number	K	Solving time	Delivery time	Solving time	Delivery time		0.56%
10	1	1.55	218	0.01	218	0.00%	
20	3	600.21	235	0.02	235	0.00%	
30	4	18,000.31	243	0.09	245	0.64%	
40	5	18,000.50	323	4.50	328	1.40%	
50	6	18,000.61	240	8.70	244	1.43%	
60	8	18,000.23	224	14.15	225	0.41%	
High-floor 20	3	700.35	337	0.60	340	0.59%	
Low-floor 20	3	600.11	147	0.04	147	0.00%	

**Table 2:** Partial Orthogonal Experiment Combinations and Total Travel Time Results.

ID	Num. of Customer Nodes	Num. of Robots	Elevator Operation Time	Total Time
1	10	1	40	245
2	20	1	50	429
3	30	1	60	362
4	40	1	70	500
5	50	1	80	725
6	60	1	90	939
7	20	2	40	235
8	30	2	50	370
9	40	2	60	519
10	50	2	70	595
11	60	2	80	500
12	60	3	90	361
13	60	4	40	295
14	50	3	50	360
15	40	5	40	328
16	30	4	40	245
17	50	5	80	239
18	60	5	90	242
19	60	6	100	245
20	50	6	40	244
21	60	7	100	240
22	40	7	80	300
23	30	8	60	230
24	50	8	70	232
25	60	8	40	225

## Data Availability

Data are contained within the article.

## Abbreviations

The following abbreviations are used in this manuscript:

MTVRP	Multi-Trip Vehicle Routing Problem (MTVRP)
ALNS	Adaptive Large Neighborhood Search
